# Are there associations between bone turnover and hip geometry in the general population?

**DOI:** 10.1016/j.afos.2025.05.007

**Published:** 2025-06-07

**Authors:** Cornelius Sebastian Fischer, Till Ittermann, Sarah Kalmbach, Moritz Herbst, Tina Histing, Jörn Lange, Anke Hannemann

**Affiliations:** a BG Klinik Tübingen, Department of Traumatology and Reconstructive Surgery, Eberhard Karls University Tübingen, Tübingen, Germany; b Institute for Community Medicine, University Medicine Greifswald, Greifswald, Germany; c Department of General and Transplant Surgery, University Hospital Tübingen, Tübingen, Germany; d Center for Orthopaedics, Trauma Surgery and Rehabilitation Medicine, University Medicine Greifswald, Greifswald, Germany; e Institute of Clinical Chemistry and Laboratory Medicine, University Medicine Greifswald, Greifswald, Germany; f DZHK (German Center for Cardiovascular Research), Partner Site Greifswald, University Medicine Greifswald, Greifswald, Germany

**Keywords:** Hip geometry, CTX, P1NP, Bone turnover, Neck-shaft angle, Center-edge angle, Alpha angle

## Abstract

**Objectives:**

While impaired bone remodeling contributes to osteoporosis and probably to osteoarthritis, the relations between bone turnover and key hip geometry measures such as center-edge angle (CE), neck-shaft angle (NSA) or alpha angle remain unknown. We here examined the presence of associations between two bone turnover markers with hip geometric measures in adults from the general population.

**Methods:**

Data from 2037 participants (50% women) in the Study of Health in Pomerania-TREND were examined. Hip geometric parameters were obtained using magnetic resonance imaging. Serum concentrations of carboxy-terminal telopeptide of Type I collagen (CTX, bone resorption) and intact amino-terminal propeptide of Type I procollagen (P1NP, bone formation) were measured to assess bone turnover.

**Results:**

In sex-specific linear regression models adjusted for age, body mass index and physical inactivity, positive associations between CTX or P1NP and CE and inverse associations with NSA were detected. The latter were restricted to men. Thus, an increase in bone formation or resorption is related to less dysplastic (both sexes). Additionally, men with more valgus hips have lower bone turnover markers. For the alpha angle, no significant association was present.

**Conclusions:**

The observed associations between bone turnover markers and hip geometry confirm the presence of relevant relations between bone properties and hip geometry. This knowledge may aid in detection of vulnerable groups with respect to osteoarthritis and fracture risk.

## 1 Introduction

Bone turnover markers are biochemical indicators that reflect the dynamic process of bone remodeling. Bone remodeling is a tightly controlled process, in which bone formation and resorption processes are coupled [1]. In the adult skeleton (around 40 years of age), both processes are balanced to maintain skeletal integrity [1]. A disturbed bone remodeling is seen for example with menopausal transition in women, intake of estrogen antagonists or estrogen deprivation [2]. It causes an increased bone turnover, which results in higher resorption and formation. Yet, the increased number of remodeling sites leads to microstructural defects and damage [3]. Menopause is also related to an uncoupling of bone remodeling, favoring resorption over formation, which causes bone loss [2]. Therefore, the measurement of bone turnover markers can give clinicians valuable information on a patient's skeletal health [1,3]. Two recommended reference markers in clinical use are C-terminal telopeptide of Type I collagen (CTX) for bone resorption and procollagen Type I N-terminal propeptide (P1NP) for bone formation [4]. These markers are useful to monitor various bone disorders, as well as to assess the effects of treatment with antiresorptive osteoporosis therapy [3]. Higher bone turnover marker levels are significantly associated with lower bone quality measured by quantitative ultrasound (QUS) and lower bone mineral density (BMD) measured by dual-energy X-ray absorptiometry (DXA) [5]. In line with this, increased bone turnover markers were observed in patients at risk of hip fracture [4,6,7].

Next to bone turnover also bone geometry, defined here as geometric parameters of the proximal femur, might be associated with hip fracture risk [[8], [9], [10], [11]]. Thalmann et al. [10], for example, discovered that the neck-shaft angle (NSA) influences fracture site and type and Gnudi et al. [9] showed that NSA and BMD are independent risk factors for hip fractures in postmenopausal women. The latter association was confirmed in men in another study [12]. Moreover, in subchondral insufficiency fractures of the femoral head, the center-edge angle (CE) was determined as prognostic factor next to BMD [11]. In a previous study, our working group revealed several associations between hip geometry and bone properties [8]. Thus, the QUS-based fracture risk was positively associated with CE and inversely related to NSA [8].

Bone geometry is also crucial for osteoarthritis (OA). Hip dysplasia, determined by CE is a well-known risk factor for OA. Consequently, patients with CEs ≤ 20° (hip dysplasia) have a higher risk for hip OA than patients with CEs > 20° [13]. Similarly, femoroacetabular impingement, as determined by the alpha angle, has been identified as an important risk factor for OA [14]. Alterations in bone turnover have also been suggested to be related to OA [[15], [16], [17], [18], [19], [20], [21]]. In 2004, Bailey et al. [15] showed a 20-fold increased subchondral bone collagen turnover of the femoral head in OA. Meanwhile, several studies investigated the association between bone turnover markers and hip OA [[16], [17], [18]] or knee OA [[19], [20], [21]].

Hip pathologies including OA and osteoporotic hip fractures are common and associated with high economic costs. A thorough knowledge on their risk factors and interactions is mandatory for prevention. As outlined above, it is evident that a disturbed bone remodeling causes osteoporosis [3], while certain alterations in hip geometry cause OA [13], and there is some evidence that a disturbed bone remodeling may be a risk factor for OA [[15], [16], [17], [18], [19], [20], [21]] and a disturbed hip geometry may be a risk factor for osteoporotic changes [[8], [9], [10], [11], [12]]. Therefore, it is of interest to examine whether bone turnover markers and hip geometry are linked. To investigate these relations and to evaluate the potential utility of the combination of bone turnover markers and imaging-derived bone parameters, we here examined the associations of CTX and P1NP with hip geometric parameters (CE, NSA and alpha angle) determined by magnetic resonance imaging (MRI) in a large sample of adults from the general population.

## 2 Methods

### 2.1 The Study of Health in Pomerania-TREND (SHIP-TREND)

SHIP-TREND is a population-based cohort study in Northeast Germany. The study region includes the cities Greifswald, Stralsund, Anklam and the surrounding communities. A representative sample of the inhabitants of the study region was drawn that included adult men and women aged 20–79 years. From this sample, 4420 men and women (response 50.1%) participated in the baseline examinations that were conducted between 2008 and 2012. Further details on study design, sampling procedures and rationale are given elsewhere [22,23].

All procedures performed in studies involving human participants were in accordance with the ethical standards of the institutional and/or national research committee and with the 1964 Helsinki declaration and its later amendments or comparable ethical standards. Written informed consent was obtained from all individual participants included in the study.

### 2.2 Study population

All 4420 SHIP-TREND baseline participants were offered a broad range of medical examinations. These included, amongst others, a computer-assisted personal interview, anthropometric measurements, blood and urine sampling and whole-body magnetic resonance imaging (MRI). About 10% (N = 467) of SHIP-participants were not offered an MRI as they were not examined in the main study center but received a brief examination in a remote study center.

**Fig. 1:**
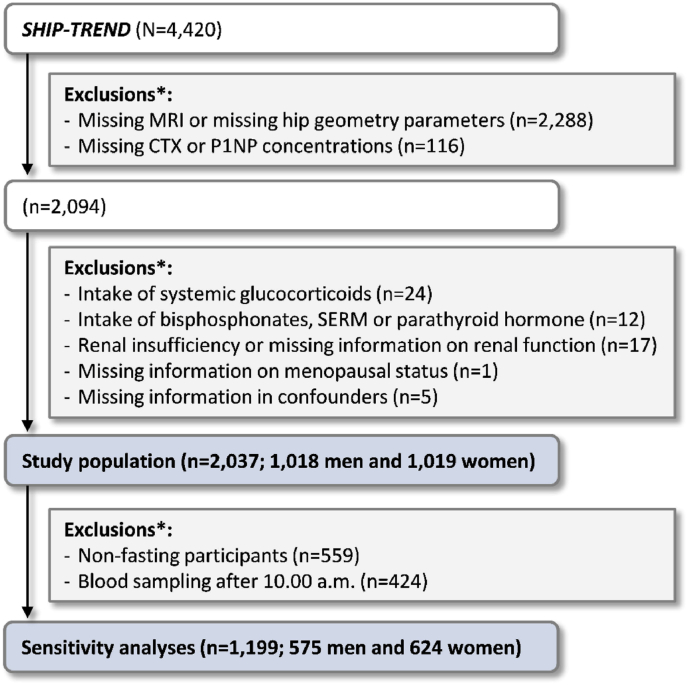
Selection of the study population. ∗Several exclusion criteria may apply to one participant. CTX, carboxy-terminal telopeptide of Type I collagen; MRI, magnetic resonance imaging; P1NP, intact amino-terminal propeptide of Type I procollagen; SERM, selective estrogen receptor modulators; SHIP-TREND, Study of Health in Pomerania-TREND.

The voluntary MRI examination could further not be performed or had to be interrupted for participants who did not consent, participants with metal implants, with claustrophobia, or acute problems during MRI. Moreover, MRIs from patients with hip arthroplasty, extreme deformity or low-quality readings could not be used for the present analyses and were defined as missing values. In total, 2288 participants without MRI or hip geometry parameters thus had to be excluded. After additionally excluding participants without CTX or P1NP measurement, 2094 subjects were available for analyses. Another 57 individuals were excluded due to intake of medication that alters bone metabolism, renal insufficiency or missing information on renal function, missing information on menopausal status or confounders. This resulted in a study population of 2037 men and women. In a sensitivity analysis, we additionally excluded all non-fasting participants (fasting for less than 8 h) and all participants with blood sampling after 10:00 a.m., which resulted in a study population of 1199 men and women (Fig. 1).

### 2.3 Measurements

Information on socio-demographic characteristics, medical histories and lifestyle were obtained by personal interviews. Physical inactivity was defined when subjects reported less than 1 h of regular physical activity per week during summer and winter. All women aged 60 years or older and all women between 40 and 60 years of age without self-reported regular menstrual cycling were considered postmenopausal, all remaining women as premenopausal. Intake of medication was recorded and classified using the anatomical therapeutic chemical classification system (ATC). Medication that alters bone metabolism was defined as glucocorticoids for systemic use (ATC-code H02AB and H02BX), bisphosphonates (ATC-code M05BA and M05BB), selective estrogen receptor modulators (ATC-code G03XC) and parathyroid hormone (ATC-code H05AA). Standardized measurements of body height and weight were performed with calibrated scales. BMI was calculated as weight (kg)/height^2^ (m^2^).

### 2.4 Laboratory methods

Venous blood samples were taken from the cubital vein of participants in the supine position. Blood sampling was performed in the mornings for the majority of samples (78%) being taken before 10:00 a.m. and the remaining samples until 12:00 a.m. About 60% of the study participants were fasting for at least 8 h before blood sampling. Serum and plasma samples were stored at −80°C in the Integrated Research Biobank (Liconic, Lichtenstein) of the University Medicine Greifswald and used in accordance with its regulations [24]. Serum CTX and P1NP concentrations were determined by automated chemiluminescent immunoassays on the IDS-iSYS Multi-Discipline Automated Analyser (Immunodiagnostic Systems Limited, Frankfurt am Main, Germany). The coefficients of variation of CTX and P1NP were 7.5% and 4.4% at low concentrations, 5.2% and 4.5% at medium concentrations, and 4.5% and 4.3% at high concentrations, respectively, of control material. Serum creatinine concentrations were determined enzymatically. The estimated glomerular filtration rate (eGFR) was calculated according to the creatinine-based Chronic Kidney Disease Epidemiology Collaboration formula [25]. Renal insufficiency was defined as eGFR < 30 ml/min/1.73 m^2^.

### 2.5 MRI protocol and angular measurements

**Fig. 2:**
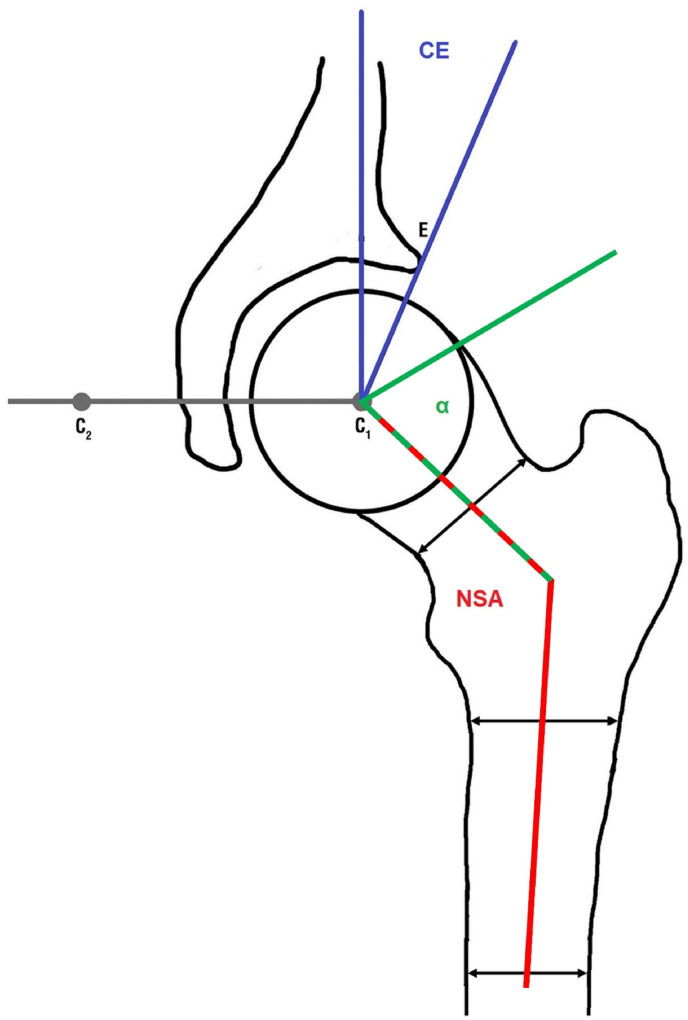
Scheme of the angular measurements.

The pelvic MRI was performed in a 1.5-T MR scanner (Magnetom Avanto; Siemens Medical Systems, Erlangen, Germany) in a supine position in a standardized manner. All measurements were performed using OsiriX version 5.8.5 (PIXMEO; Bernex, Switzerland). The observer was blinded to all information about the participants. The CE, NSA and alpha angle were measured on a coronal planar image while the center of the femoral head was identified by using axial slices simultaneously (Fig. 2). Detailed information on the sequence specifics and measurements were published previously [26,27].

The center-edge angle (CE) is composed by a line perpendicular to the connection line between each femoral head center (C1 und C2) and a line through the lateral acetabular margin (E). The alpha angle (α) is composed by the femoral neck axis and the line through the point where the contour of the femoral head-neck junction exceeds the radius of the femoral head. The neck-shaft angle (NSA) is formed by the femoral neck axis and the femoral shaft axis.

### 2.6 Statistical analyses

General characteristics of the study population, stratified by sex, are given as medians with 1st-3rd quartiles (continuous data) or as proportions (nominal data). Sex-differences were tested with χ² or Kruskal-Wallis tests and P-values < 0.05 were considered statistically significant.

In separate linear regression models, associations between the two bone turnover markers (exposures) and the hip geometry parameters (outcomes) were assessed. CTX and P1NP were log-transformed before being entered in the regression models. Thus, the effects of a one unit increase in log-transformed CTX or P1NP on CE, NSA and alpha angle were examined. To account for multiple testing, we adjusted the P-values by controlling the false discovery rate (FDR) at 5% using the Benjamini-Hochberg procedure [28]. We then examined whether effect modification by sex, age, or menopausal status (in women only) was present. In our regression models adjusted for sex, age, BMI and physical inactivity, interaction terms for sex were significant in five out of six models (FDR < 0.05). Interaction terms for menopausal status were not statistically significant (FDR < 0.05). This indicates that the effects of the bone turnover markers on CE, NSA and alpha angle do not vary substantially between pre- and postmenopausal women. The resulting main models were therefore calculated separately in men and women and adjusted for age, BMI and physical inactivity. We report β-coefficients with standard errors or 95% confidence intervals, P-values and FDRs from these models.

Bone turnover markers, especially bone resorption markers, underly a circadian rhythm and are affected by fasting status [29]. In a first sensitivity analysis, we therefore recalculated all models after exclusion of non-fasting participants and participants with blood sampling after 10:00 a.m. In a second sensitivity analysis, we examined the effects of a potential selection bias introduced by the large number of excluded participants. As mentioned above, the study population was selected from the subgroup of SHIP-TREND participants with whole-body MRI. These individuals were free of contraindications for MRI, eg, metal implants, and therefore probably healthier than the SHIP-TREND population in general. We generated inverse probability weights based on sex, age, BMI, smoking status, alcohol consumption, self-reported physician's diagnosis of hypertension, and physical inactivity as explanatory variables, as these variables are assumed to affect MRI participation. We then recalculated our main model weighted for non-participation in MRI to examine the stability of the results.

All statistical analyses were performed with SAS 9.4 (SAS Institute Inc., Cary, NC, USA).

## 3 Results

Men (N = 1018) and women (N = 1019) were equally represented in our study population and had an equal median age of 51 years. More than half of the women were postmenopausal. The average study participant was overweight (median BMI of 27.8 kg/m^2^ in men and 26.3 kg/m^2^ in women) and about one third of men and women were physically inactive. The concentration of the bone resorption marker CTX was higher in men than in women while the concentration of the bone formation marker P1NP was comparable between the sexes. Regarding hip geometry parameters, CE and NSA were on average higher in women than in men, while alpha angle was higher in men than in women (Table 1).

**Table 1:** Characteristics of the study population separated by sex.

Characteristics	Men (N = 1018)	Women (N = 1019)	P
Age, yrs	51.0 (41.0–63.0)	51.0 (41.0–61.0)	0.43
Postmenopausal, %	–	56.3	–
BMI, kg/m^2^	27.8 (25.4–30.5)	26.3 (23.3–30.2)	<0.01
Physical inactivity, %	30.4	28.5	0.35
eGFR, ml/min/1.73m^2^	97.6 (87.4–107.8)	97.5 (85.8–107.0)	0.31
Fasting, %	68.3	76.8	<0.01
Blood sampling before 10:00 a.m., %	80.2	78.2	0.28
CTX, ng/ml	0.26 (0.17–0.39)	0.23 (0.15–0.37)	<0.01
P1NP, ng/ml	44.4 (34.1–57.0)	44.1 (33.5–58.1)	0.83
Center-edge angle, °	30.2 (25.4–35.2)	32.0 (27.4–36.8)	<0.01
Neck-shaft angle, °	127.4 (123.0–131.5)	128.1 (123.7–132.7)	<0.01
Alpha-angle, °	56.3 (51.1–63.3)	50.8 (47.0–55.1)	<0.01

Data are proportions or medians (1st-3rd quartile). Group differences were tested with χ^2^-tests (categorical data) or Kruskal-Wallis tests (continuous data).

BMI, body mass index; CTX, carboxy-terminal telopeptide of Type I collagen; eGFR, estimated glomerular filtration rate; P1NP, intact amino-terminal propeptide of Type I procollagen.

**Fig. 3:**
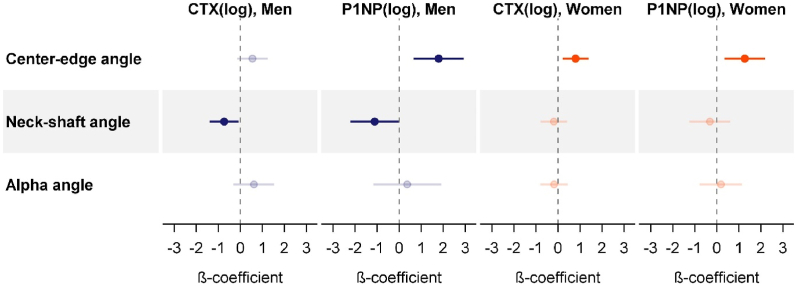
Sex-specific associations between the two bone turnover markers, CTX and P1NP, and the hip geometry parameters. Illustrated are the β-coefficients with 95% confidence intervals of a one unit increase in log-transformed CTX and P1NP from linear regression models. Results for men are colored in blue, results for women are colored in orange. Bright colors illustrate statistically significant associations after correction for multiple testing (FDR <0.05). All models were adjusted for age, BMI and physical inactivity. BMI, body mass index; CTX, carboxy-terminal telopeptide of Type I collagen; FDR, false discovery rate; P1NP, intact amino-terminal propeptide of Type I procollagen. (For interpretation of the references to color in this figure legend, the reader is referred to the Web version of this article.)

Multivariable regression analyses revealed some general findings (Fig. 3 and Table 2). First, there was no association between the bone turnover markers and alpha-angle, neither in men nor in women. Second, associations between the bone turnover markers and CE were generally positive, while associations with NSA were inverse. Third, the effects of CTX and P1NP point in the same direction, ie, higher bone resorption had the same effect on hip geometry as higher bone formation.

**Table 2:** Sex-specific associations between the two bone turnover markers, CTX and P1NP, and the hip geometry parameters.

Exposure	Outcome	Main Analysis					Sensitivity Analysis				
		β	stderr	95% CI	P	FDR	β	stderr	95% CI	P	FDR
P1NP(log), Men	Center-edge angle	1.79	0.58	0.65; 2.93	0.002	0.004	1.29	0.75	−0.18; 2.75	0.085	0.169
	Neck-shaft angle	−1.12	0.56	−2.22; −0.02	0.046	0.046	−1.93	0.75	−3.40; −0.46	0.010	0.020
	Alpha angle	0.36	0.79	−1.18; 1.90	0.643	0.643	0.71	1.07	−1.39; 2.81	0.506	0.506
CTX(log), Men	Center-edge angle	0.55	0.35	−0.14; 1.23	0.117	0.117	0.62	0.50	−0.36; 1.61	0.216	0.216
	Neck-shaft angle	−0.74	0.33	−1.40; −0.09	0.026	0.046	−1.10	0.50	−2.09; −0.11	0.029	0.029
	Alpha angle	0.61	0.47	−0.31; 1.53	0.194	0.387	0.69	0.72	−0.72; 2.10	0.335	0.506
P1NP(log), Women	Center-edge angle	1.27	0.47	0.36; 2.18	0.007	0.008	0.83	0.59	−0.32; 1.98	0.158	0.158
	Neck-shaft angle	−0.32	0.48	−1.25; 0.61	0.503	0.535	−0.64	0.60	−1.82; 0.54	0.289	0.577
	Alpha angle	0.19	0.49	−0.77; 1.15	0.702	0.702	0.04	0.59	−1.13; 1.20	0.951	0.951
CTX(log), Women	Center-edge angle	0.80	0.30	0.21; 1.39	0.008	0.008	1.04	0.40	0.26; 1.82	0.009	0.018
	Neck-shaft angle	−0.19	0.31	−0.79; 0.41	0.535	0.535	−0.18	0.41	−0.99; 0.62	0.654	0.654
	Alpha angle	−0.18	0.32	−0.80; 0.44	0.569	0.702	−0.15	0.40	−0.95; 0.64	0.704	0.951

β-coefficients with standard errors and 95% confidence intervals (CI) for a one unit increase in log-transformed CTX and P1NP from linear regression models. All models were adjusted for age, BMI and physical inactivity. Statistically significant associations after correction for multiple testing (FDR < 0.05) are printed in bold.

BMI, body mass index; CTX, carboxy-terminal telopeptide of Type I collagen; FDR, false discovery rate; P1NP, intact amino-terminal propeptide of Type I procollagen.

**Fig. 4:**
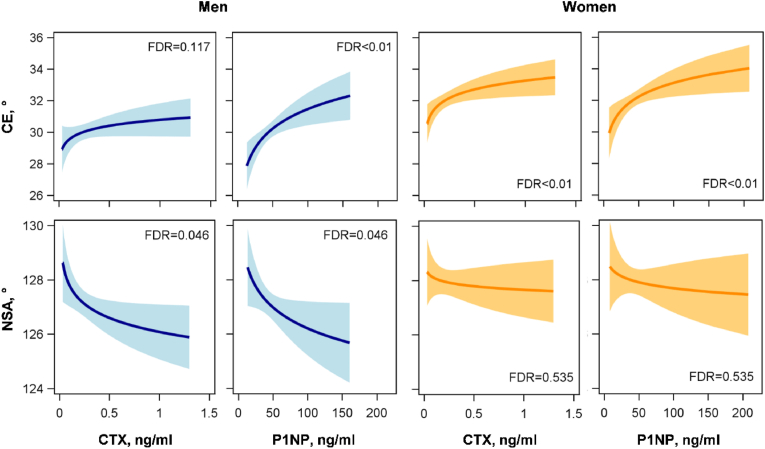
Associations between CTX or P1NP and center-edge angle (CE) or neck-shaft angle (NSA) in men (N = 1018) and women (N = 1019). Illustrated are the regression lines (solid black line) with 95% confidence interval from the fully adjusted models for an average individual (age = 51 years, BMI = 27 kg/m^2^, physically active). BMI, body mass index; CTX, carboxy-terminal telopeptide of Type I collagen; FDR, false discovery rate; P1NP, intact amino-terminal propeptide of Type I procollagen.

In detail, increasing CTX concentrations in women and increasing P1NP concentrations in men and women were related to higher CEs. The association between CTX and CE in men was also positive, but missed statistical significance. The inverse associations between CTX or P1NP with NSA were, on the other side, restricted to men and missed statistical significance in women (Fig. 3, Fig. 4 and Table 2).

The results of our first sensitivity analysis generally confirmed the main findings (Table 2), albeit with slightly altered effects. The exclusion of non-fasting participants and participants with blood sampling after 10:00 a.m. reduced the sample size dramatically. This resulted in increased standard errors and confidence intervals, which rendered the association between P1NP and CE in men and women statistically insignificant. Nevertheless, the observed effects remained quite similar. Moreover, the effect direction and statistical significance observed between P1NP and NSA in men remained stable in this analysis.

The results of our second sensitivity analysis also confirmed our main results. In the models weighted for non-participation, all statistically significant associations and their directions from the main analysis remained present and the effect sizes changed only slightly (Supplemental Table 1).

## 4 Discussion

Combining information on bone turnover and hip geometry might be valuable for the identification of patients at risk for hip pathologies, in case associations between these measures exists. The present study investigated associations between two well-known bone turnover markers and hip geometry in a large sample of the population-based SHIP-TREND study. In sex-specific analyses, significant positive associations between CTX or P1NP and CE (both sexes) and inverse associations between the bone turnover markers and NSA (restricted to men) were detected. Associations with alpha angle were absent.

As our society is aging, proximal femur fractures and OA are an increasing socioeconomic challenge. Since increased subchondral bone turnover was detected in OA joints [19], several studies examined and confirmed bone turnover markers as predictors for OA. Urinary alpha CTX as well as urinary CTX-II, for example, were determined as non-invasive markers and predictors for joint remodeling and pain worsening in knee OA [19,30]. Bihlet et al. [31] confirmed the association for uCTX-II, whereas only trends and no significant association were detected for uCTX-I or osteocalcin. In addition, Chen et al. [21] suggested a combination of parathyroid hormone and β-CTX for the identification of OA in high-risk men. For hip OA, associations of CTX-II with pain and radiographic signs of joint damage were revealed [16]. Multiple studies [18,32,33] as well as the meta-analysis of Valdes et al. [34] therefore suggested CTX-II as a sensitive quantitative marker for OA. Regarding P1NP, opposing results have been presented. Watanabe et al. [17], for example, discovered higher levels of P1NP in patients with rapidly destructive coxarthrosis. Moreover, P1NP was associated with joint space narrowing in patients with subchondral insufficiency fractures of the femoral head, and therefore suggested as predictor for OA [35]. Several other studies, in contrast, found no association between OA and serum P1NP or CTX-I [18,36]. Garnero et al. [18], for instance, demonstrated that serum CTX-I and P1NP are specific systemic bone turnover markers and not specific cartilage markers, while urinary CTX-II represent an independent risk factor for total joint replacement. Since hip dysplasia is a generally accepted risk factor for OA [13], and might also relate to hip fracture risk or BMD [[9], [10], [11], [12]], knowledge on associations with bone turnover markers is of interest. To our knowledge, this is the first study to examine these relations in adult humans. It found, that increasing serum CTX and P1NP concentrations were related to less dysplastic hips, while no association was present with the alpha angle. This aligns with previous results from our working group, demonstrating positive associations of CE with bone stiffness index [8].

Bone turnover markers may assist in evaluating a patients’ bone health. Several studies documented associations between bone turnover markers and BMD [20] as well as bone quality assessed by QUS [5]. Normal fracture healing leads to increased bone turnover markers [1,37,38]. Resmini et al. [39] documented significant differences in bone turnover markers for fracture-patients undergoing hip prosthesis compared to those operated for OA. Regarding fracture type, Iizuka et al. [40] showed significant lower P1NP in patients with atypical femur fractures compared to typical femur fractures. Various further studies described an increased fracture risk in patients with increased CTX and P1NP [[41], [42], [43]]. These associations were independent from BMD in postmenopausal women [4,7]. Lou et al. [44] concluded that altered BMD, P1NP, β‐CTX and vitamin D concentrations might help to identify high‐risk individuals for hip fractures among osteoporotic elderly patients. Since hip geometry influences fracture risk [9,10], information on possible associations with bone turnover markers might be valuable. Liu et al. [45] determined that NSA together with the osteocalcin level are important risk factors for fractures in patients with fibrous dysplasia. The present study determined an inverse association between CTX or P1NP and NSA in men. Combined with previous results from our working group [8], these data indicate that individuals with more valgus hips have lower bone turnover markers concentrations and a lower fracture risk. This observation is in accordance with previous reports, that suggested lower NSA in fracture cases [46]. In the last decades, identification of vulnerable patients for hip fractures improved, eg, due to the measurement of BMD and the implementation of fracture risk assessment tools, eg, FRAX [47]. The results of this study indicate a relation between hip geometry and bone turnover, which could have implications for fracture risk, eg, increase fracture risk above that of each risk factor alone. While this hypothesis could not be tested in the current study, it poses a promising field for future research.

The present study has several limitations. Our results were obtained in adults form the general population. Yet, it should be noted that a significant proportion of individuals were either not offered or refused whole-body MRI or were not eligible for the examination due to contraindications, eg, participants with metal implants. Consequently, our study population may be slightly healthier than the general population. In a sensitivity analysis we applied inverse probability weight for non-participation in the whole-body MRI and were able to rule out a possible bias due to participant selection. We therefore assume, that our results are valid for the general population in our study region. Another limitation is the evaluation of the hip geometry using non-rotation corrected, two-dimensional coronal MRI. However, as previously noted [26,27], the presented values align with those from other acquisition methods and can be considered reliable. The third limitation is the cross-sectional study design, which allows the identification of associations, but does not establish cause-and-effect relationships. Beside these limitations, our study stands out due to the large number of participants, that allowed us to perform sex-specific analyses. Furthermore, the highly standardized and extensive data collection ensures a high data quality and allowed us to adjust our models for interfering covariates. Moreover, our results were confirmed after additionally excluding non-fasting participants and those with blood sampling in the afternoon, conditions that are known to bone turnover markers concentrations, especially CTX.

## 5 Conclusions

This study demonstrates that, in adult men and women, several associations between bone turnover and hip geometry are present. The positive association between CTX as well as P1NP and CE suggests that individuals with less dysplastic hips have higher bone turnover markers concentrations. In addition, the inverse associations of the bone turnover markers and NSA suggests that men with more valgus hips have lower bone turnover markers. We therefore suggest further studies to examine whether the relations between bone turnover and hip geometry exert joint effects on (patho)-physiologic changes preceding osteoporosis and OA. This knowledge may aid in the detection of vulnerable groups with respect to both entities.

## CRediT author statement

**Cornelius Sebastian Fischer**: Conceptualization, Data curation, Investigation, Methodology, Project administration, Supervision, Writing - original draft. **Till Ittermann**: Validation, Software, Writing - review & editing. **Sarah Kalmbach**: Writing - review & editing. Moritz Herbst: Writing - review & editing. **Tina Histing**: Writing - review & editing. **Jörn Lange**: Writing - review & editing. **Anke Hannemann**: Conceptualization, Data curation, Formal analysis, Methodology, Project administration, Software, Visualization, Writing - original draft.

## Conflicts of interest

The authors declare no competing interests.
