# Supplementary material for: Are there associations between bone turnover and hip geometry in the general population?
Source: Osteoporos Sarcopenia. 2025 Jun 7;11(2):50–6. doi: 10.1016/j.afos.2025.05.007 (PMC12266172; doi:10.1016/j.afos.2025.05.007)
Supplement: Multimedia component 1 [file mmc1.docx]

**Supplemental Material**

**Supplemental Table 1.** Sex-specific associations between the two bone turnover markers, CTX and P1NP, and the hip geometry parameters in weighted models.

| **Exposure** | **Outcome** | **ß** | **stderr** | **95% CI** | **P** | **FDR** |
| --- | --- | --- | --- | --- | --- | --- |
| CTX(log), Men | Center-edge angle | 0.54 | 0.35 | -0.14; 1.22 | 0.118 | 0.118 |
|  | Neck-shaft angle | -0.67 | 0.33 | -1.32; -0.02 | 0.044 | 0.045 |
|  | Alpha angle | 0.54 | 0.46 | -0.37; 1.45 | 0.244 | 0.487 |
| P1NP(log), Men | Center-edge angle | 1.75 | 0.59 | 0.60; 2.90 | 0.003 | 0.006 |
|  | Neck-shaft angle | -1.13 | 0.56 | -2.23; -0.02 | 0.045 | 0.045 |
|  | Alpha angle | 0.36 | 0.78 | -1.17; 1.90 | 0.644 | 0.644 |
| CTX(log), Women | Center-edge angle | 0.81 | 0.30 | 0.22; 1.40 | 0.007 | 0.007 |
|  | Neck-shaft angle | -0.21 | 0.31 | -0.82; 0.39 | 0.493 | 0.494 |
|  | Alpha angle | -0.21 | 0.31 | -0.82; 0.41 | 0.513 | 0.879 |
| P1NP(log), Women | Center-edge angle | 1.28 | 0.47 | 0.36; 2.20 | 0.006 | 0.007 |
|  | Neck-shaft angle | -0.33 | 0.48 | -1.27; 0.61 | 0.494 | 0.494 |
|  | Alpha angle | 0.07 | 0.49 | -0.88; 1.03 | 0.879 | 0.879 |

ß-coefficients with standard errors (stderr) and 95% confidence intervals (CI) for a one unit increase in log-transformed CTX and P1NP from linear regression models. All models were adjusted for age, BMI and physical inactivity and weighted for non-participation in whole-body MRI. The inverse probability weights were generated using sex, age, BMI smoking, alcohol consumption, hypertension, and physical inactivity as explanatory variables. Statistically significant associations after correction for multiple testing (FDR < 0.05) are printed in bold.

BMI, body mass index; CTX, carboxy-terminal telopeptide of type I collagen; FDR, false discovery rate; MRI, magnetic resonance imaging; P1NP, intact amino-terminal propeptide of type I procollagen.
